# LRP5-Mediated Lipid Uptake Modulates Osteogenic Differentiation of Bone Marrow Mesenchymal Stromal Cells

**DOI:** 10.3389/fcell.2021.766815

**Published:** 2021-11-02

**Authors:** Jiachen Lin, Zhifa Zheng, Jieying Liu, Guihua Yang, Ling Leng, Hai Wang, Guixing Qiu, Zhihong Wu

**Affiliations:** ^1^Medical Science Research Center, State Key Laboratory of Complex Severe and Rare Diseases, Peking Union Medical College Hospital, Chinese Academy of Medical Sciences and Peking Union Medical College, Beijing, China; ^2^Department of Orthopedic Surgery, Peking Union Medical College Hospital, Chinese Academy of Medical Sciences and Peking Union Medical College, Beijing, China; ^3^Harmony Technology Co., Ltd., Beijing, China

**Keywords:** bone marrow mesenchymal stromal cell (BMSC), low-density lipoprotein receptor-related protein 5 (LRP5), osteogenic differentiation, lipid metabolism, conditional knockout mice

## Abstract

Nutritional microenvironment determines the specification of progenitor cells, and lipid availability was found to modulate osteogenesis in skeletal progenitors. Here, we investigated the implications of lipid scarcity in the osteogenic differentiation of bone marrow mesenchymal stromal cells (BMSCs) and the role of low-density lipoprotein receptor-related protein 5 (LRP5), a co-receptor transducing canonical Wnt/beta-catenin signals, in BMSC lipid uptake during osteogenesis. The osteogenic differentiation of murine BMSCs was suppressed by lipid scarcity and partially rescued by additional fatty acid treatment with oleate. The enhancement of osteogenesis by oleate was found to be dosage-dependent, along with the enhanced activation of beta-catenin and Wnt target genes. Conditional knockout (CKO) of *Lrp5* gene in murine mesenchymal lineage using *Lrp5^*fl/fl*^;Prrx1-cre* mice led to decreased bone quality and altered fat distribution *in vivo*. After *Lrp5* ablation using adenoviral Cre-recombinase, the accumulation of lipid droplets in BMSC cytoplasm was significantly reduced, and the osteogenesis of BMSCs was suppressed. Moreover, the impaired osteogenesis due to either lipid scarcity or *Lrp5* ablation could be rescued by recombinant Wnt3a protein, indicating that the osteogenesis induced by Wnt/beta-catenin signaling was independent of LRP5-mediated lipid uptake. In conclusion, lipid scarcity suppresses BMSC osteogenic differentiation. LRP5 plays a role in the uptake of lipids in BMSCs and therefore mediates osteogenic specification.

## Introduction

Nutritional microenvironment determines the specification of skeletal progenitor cells. As one of the essential metabolites in mammals, lipids serve not only as important fuel sources but also as biological mediators for the proper functioning and homeostasis of bone (Rendina-Ruedy and Rosen, 2020). Recently, lipids were revealed as key modulators to the cell fate of multiple skeletal lineages, including periosteal cells, osteoblasts, and chondrocytes, during the vascularization and regeneration after bone fracture (van Gastel et al., 2020). Bone marrow mesenchymal stromal cells (BMSCs) harbor a subpopulation of skeletal progenitors with potency to undergo osteogenesis (Yue et al., 2016; Chan et al., 2018; Baccin et al., 2020) and are therefore considered favorable seeding cells in many bone regenerative investigations (Lv et al., 2017; Mazzoni et al., 2020; Ying et al., 2020). However, the regulatory role of lipids in the osteogenic differentiation of BMSCs has not yet been elucidated.

Low-density lipoprotein (LDL) receptor-related protein 5 (LRP5) is a member of LDL receptor family and also known as a co-receptor for canonical Wnt/beta-catenin signaling pathway and transduces key molecular signals in bone development and homeostasis (Cui et al., 2011; Baron and Kneissel, 2013; Kobayashi et al., 2016). Several newly uncovered molecular mechanisms about Wnt-independent LRP5 modulation in bone (Clement-Lacroix et al., 2005; Yadav and Ducy, 2010; Chin et al., 2015; Frey et al., 2015; He et al., 2020) suggested a more complicated role of LRP5 in skeletal progenitors. Notably, recognized as a cell surface endocytic receptor (Li et al., 2001; Schneider and Nimpf, 2003; Chung and Wasan, 2004), LRP5 was also found to participate in the uptake and internalization of lipids in macrophages (Badimon et al., 2020), hepatocytes and adrenal cortex tissues (Kim et al., 1998), and osteoblasts (Frey et al., 2015). There are other studies indicating the modulatory role of LRP5 in lipid metabolism (Magoori et al., 2003; Loh et al., 2015).

In order to investigate the specific role of lipid availability and LRP5 in the osteogenic differentiation of BMSCs, we here carried out *in vivo* and *in vitro* assays using BMSCs isolated from wild-type mice and *Lrp5* gene conditional-ready (i.e., *Lrp5*-floxed) mice. We recorded the suppression of osteogenesis and Wnt-target genes in BMSCs by lipid scarcity and a dosage-dependent rescue of the phenotype using oleate, an unsaturated fatty acid, as a supplement. The *Lrp5^*fl/fl*^;Prrx1-cre* CKO mice presented with impaired bone formation and altered fat distribution. Moreover, we observed a significant decrease in lipid uptake and suppressed osteogenesis of *Lrp5*^*fl/fl*^ BMSCs after adenoviral Cre-recombination, and it was rescued by additional recombinant Wnt3a protein. In summary, these findings suggested that lipid scarcity suppressed osteogenic differentiation of BMSCs, and LRP5-mediated lipid uptake could modulate osteogenic differentiation of BMSCs.

## Materials and Methods

### Mouse Lines and Genotyping

This study was approved by the Ethics Committee of Peking Union Medical College Hospital. C57BL/6J mice were obtained from the Vital River Laboratory Animal Technology (Beijing, China). *Lrp5*^*fl/fl*^ conditional-ready mice and *Prrx1-cre* transgenic mice were purchased from the Shanghai Model Organisms Center (Shanghai, China). To generate *Lrp5* CKO mice, *Lrp5*^*fl/fl*^ mice were crossed with *Prrx1-cre* mice to get *Lrp5^*fl/fl*^; Prrx1-cre* genotype. Mouse genome DNA was extracted from tail of mice at postnatal day 10 (P10), and the amplification of target genes (*Lrp5* and *Prrx1-cre*) was done following a described protocol (Truett et al., 2000; Cui et al., 2011). A 2.5% Agarose-TAE gel containing Gel-Green (D0143, Beyotime, Beijing, China) was used for electrophoresis of PCR products.

### Cell Culture

Murine BMSCs were obtained using a previous described protocol with minor modifications (Grassel et al., 2012). In brief, murine femur and humerus were dissected from *Lrp5*^*fl/fl*^ or wild-type C57BL/6J mice at P14 after euthanasia and washed three times in cold phosphate buffered saline (PBS; Cytiva) with 1% penicillin-streptomycin (Sigma-Aldrich). The isolated long bones were then cut with sterile scissors, and the bone marrow was extracted by centrifuging at 500 *g* for 30 s. Pellet was resuspended in PBS and filtered with 70-μm cell strainer (BD Falcon). Cells were then resuspended and seeded in 10-cm dishes (Corning) in Dulbecco’s modified Eagle’s medium (DMEM) high glucose (Hyclone), 1% penicillin-streptomycin (Sigma-Aldrich), and 10% fetal bovine serum (FBS; Gibco). BMSCs were cultured at 37°C and 5% CO_2_, and the cell medium was changed every 48 h. After 80–90% cell confluence was reached, the adherent cells were washed twice in PBS, trypsinized using 0.25% Trypsin-EDTA (Thermo Fisher Scientific), centrifuged at 1,000 *g* for 3 min, and then seeded in 10-cm dish (Corning) at the next passage.

### Cell Viability

Cell viability was determined by the methylthiazolyldiphenyl-tetrazolium bromide (MTT) assay as described previously (Riss et al., 2004). In brief, MTT solution was added to each well 7 days after osteogenic induction with mediums of different lipid treatment. Three hours later, dimethyl sulfoxide (DMSO) was added and the absorbance of the solution was measured at 570 nm with an automatic microplate reader (CFX Connect, Bio-Rad). The survival rate of cells in percentage was calculated using DMSO-treated cells as a standard. One-way ANOVA was conducted with GraphPad Prism software.

### Induction of Osteogenic Differentiation

The osteogenic differentiation of BMSCs was induced as described previously (Grassel et al., 2012). The third-passage BMSCs were seeded in 0.1% gelatin (Sigma-Aldrich) pretreated 6- or 12-well plates (Corning) at 5,000 cells/100 μl. The osteogenic induction was initiated at 70–80% cell confluence by using an osteogenic medium composed of DMEM high glucose (Hyclone), 100 nM dexamethasone (Sigma-Aldrich), 0.05 mM ascorbate 2-phosphate (Sigma-Aldrich), 10 mM sodium beta-glycerophosphate (Sigma-Aldrich), and 1% penicillin-streptomycin (Sigma-Aldrich). FBS (Gibco), lipid-reduced serum (Biowest), oleate (Sigma-Aldrich), and recombinant Wnt3a protein (5036-WN, R&D Systems) were added into the medium according to the experiment design.

### Assessment of Alkaline Phosphatase Activity

After 5/7/11 days of osteogenic induction, the osteogenic differentiation was assessed by alkaline phosphatase (ALP) staining kit (VectorLab) in the 12-well plate. After 30 min staining, the BMSCs were fixed in 4% paraformaldehyde (P1110, Solarbio, Beijing, China), washed in PBS (Cytiva). Then, the 12-well plate was scanned and photographed under × 4 magnification using EVOS Cell Imaging System (EVOS M7000, Thermo Fisher Scientific). The ALP activity of each well was quantified with ImageJ Software (^1^ National Institutes of Health) as previously described (Dupont et al., 2011). In essence, the ALP + area was determined with ImageJ as the number of blue pixels across the picture, and then this value was normalized to the total number of pixels for each picture. For each well, views of four different zones were adopted for calculations. Multiple *t* tests were conducted with GraphPad Prism software using the Holm–Sidak test.

### Fluorescent Staining of Lipid Droplets in Cytoplasm

Bone marrow mesenchymal stromal cells were seeded in six-well plate under different conditions. BODIPY^TM^ staining kit (Thermo Fisher Scientific) was applied to trace the endocytosis of lipid substance in BMSCs (DiDonato and Brasaemle, 2003), and 4,6-diamidino-2-phenylindole staining solution (C00065, Solarbio, Beijing, China) was used to locate the nucleus. After BODIPY^TM^ application, the plate was scanned and photographed under × 40 magnification using EVOS Cell Imaging System (EVOS M7000, Thermo Fisher Scientific). Cells with positive signals in cytoplasm were counted, and the fluorescence intensity was calculated by BODIPY^TM^-positive cell counts over total cell in the region. Statistical comparisons were conducted with GraphPad Prism software using the Holm–Sidak test. For each analysis, four different views were adopted to carry out the quantification.

### Adenoviral Vector Preparation and Infection

Adenoviruses expressing CMV-Cre-recombinase and CMV-Null vectors were purchased from Beyotime (Beijing, China). BMSCs were infected with viruses at 100 multiplicity of infection (MOI) in the presence of 0.1% poly-L-lysine (Sigma-Aldrich) for 1 h as previously described (Buo et al., 2016).

### RNA Extraction, Reverse Transcription, and Real-Time PCR

Total protein was extracted from BMSC cell lysates, murine bone tissue, and other organs using TRIzol (Thermo Fisher Scientific) following the standard protocols. cDNA was obtained using PrimerScript RT (TaKaRa, Japan). Real-time PCR was performed using TB Green Master Mix kit (TaKaRa, Japan) on Applied Biosystems StepOnePlus Real-Time PCR System (Thermo Fisher Scientific). The primers used for qPCR analysis were listed in the Supplementary Table. mRNA expression levels of *Alpl*, *Sp7*, *Col1a1*, *Tcf1*, *Lef1*, and *Axin2* were normalized to *glyceraldehyde 3-phosphate dehydrogenase* (*Gapdh*). Statistical comparisons were conducted with GraphPad Prism software using the Holm–Sidak test. Each experiment was repeated three times.

### Protein Extraction and Western Blotting

Total protein was extracted from BMSC cell lysates, murine bone tissue, and other organs using radioimmunoprecipitation assay (RIPA) buffer (Thermo Fisher Scientific). The protein concentration was determined by bicinchoninic acid (BCA) protein assay kit (Thermo Fisher Scientific). Western blot was performed using a standard protocol. Anti-LRP5 (rabbit monoclonal, ab223203, Abcam, 1:1,000), anti-Type I Collagen (COL1; rabbit monoclonal, ab270993, Abcam, 1:1,000), anti-RUNX Family Transcription Factor 2 (RUNX2; rabbit monoclonal, ab236639, Abcam, 1:1,000), anti-beta catenin (rabbit monoclonal, ab32572, Abcam, 1:5,000), and anti-GAPDH (rabbit monoclonal, #2118, Cell Signaling Technology, 1:1,000) were applied as primary antibodies, and horseradish peroxidase (HRP) goat anti-rabbit immunoglobulin G (IgG) (H + L) (ZB-2301, ZSGB Biotech, Beijing, China, 1:2,000) was applied as secondary antibody to carry out immunoblotting. Protein expression levels were visualized by automatic chemiluminescence imaging system (C300, Azure Biosystems). The quantification of band intensity was performed by ImageJ Software. Overall expression levels of LRP5, COL1, RUNX2, and beta-catenin were normalized to GAPDH levels. Statistical comparisons were conducted with GraphPad Prism software using the Holm–Sidak test. Three repetitions were carried out for each of the above experiments.

### Micro-Computed Tomography and Assessment of Bone Quality

Control and CKO mice at P21 were euthanized. Femora and humeri were obtained and fixed in 4% paraformaldehyde (P1110, Solarbio, Beijing, China) for 48 h, washed in cold PBS three times, and then preserved in 75% ethanol at 4°C. Samples were scanned and analyzed using SCANCO micro-computed tomography (micro-CT) μ100 system (SCANCO Medical). The three-dimensional structural parameters including humerus length, mean bone density, trabecular bone volume fraction (Tb.BV/TV), average cortical thickness (Ct.Th), trabecular number (Tb.N), and trabecular separation (Tb.Sp) were analyzed to determine the bone quality as previously described (Bouxsein et al., 2010). Each analysis was performed in samples collected from 12 individual animals (six control mice and six CKO mice). Statistical comparisons were conducted with GraphPad Prism software using unpaired *t*-test.

### Dissection of Adipose Tissue

Here, 2-month-old control or CKO mice were euthanized. Intact adipose tissues in gonadal, mesenteric, and subcutaneous regions were carefully dissected using forceps and scissors. The percentage of fat pad mass (g) over body weight (g) was calculated. Each analysis was repeated with 12 individual animals (six control mice and six CKO mice). Statistical comparisons were conducted with GraphPad Prism software using the Holm–Sidak test.

### Statistical Analysis

All data analysis in this study was carried out on GraphPad Prism 8 (GraphPad Software). At least three independent experimental groups were performed to generate a qualified data set. Statistical strategies adopted in each experiment were described above. *P*-values < 0.05 (^∗^), 0.01 (^∗∗^), and 0.001 (^∗∗∗^) were considered significant. Error bars on all graphs are presented as the standard deviation of the mean unless otherwise indicated.

## Results

### Lipid Scarcity Suppressed Osteogenic Differentiation of Murine Bone Marrow Mesenchymal Stromal Cells and Wnt/Beta-Catenin Signaling

**FIGURE 1:**
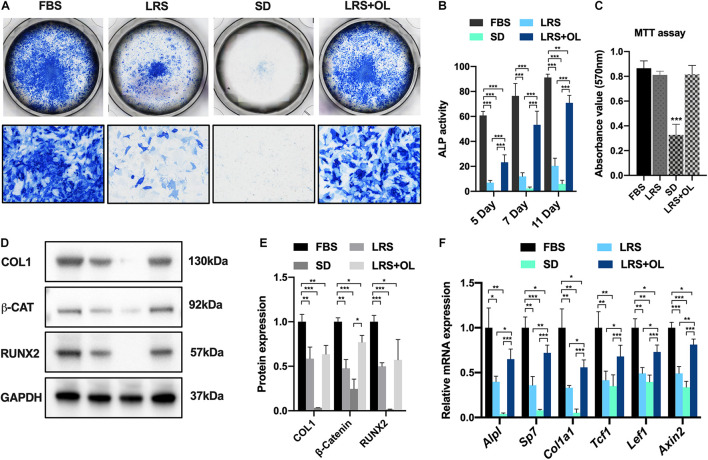
Lipid scarcity suppressed bone marrow mesenchymal stromal cell (BMSC) osteogenesis and Wnt/beta-catenin signaling activation. **(A)** Alkaline phosphatase (ALP) staining of BMSCs 7 days after osteogenic induction in culture medium with normal fetal bovine serum (FBS; 10%), lipid-reduced serum (LRS; 10%), SD, and LRS (10%) with 75 μM oleate treatment. **(B)** ALP activity of BMSCs 5, 7, and 11 days after osteogenic induction in different culture mediums. **(C)** Methylthiazolyldiphenyl-tetrazolium bromide (MTT) assays revealed significantly decreased cell viability in SD treatment BMSCs compared to FBS treatment group, while the cell viability was not significantly changed in LRS and LRS + OL group. **(D,E)** Western blots showed that the protein expression levels of osteogenic markers Type I Collagen (COL1) and RUNX Family Transcription Factor 2 (RUNX2) and beta-catenin were significantly decreased after lipid deprivation and partially restored by additional oleate treatment 7 days after induction. **(F)** qRT-PCR revealed that the mRNA expression levels of osteogenic genes, *Alpl*, *Sp7*, and *Col1a1*, as well as Wnt target genes *Tcf1*, *Lef1*, and *Axin2*, were significantly decreased after lipid deprivation and partially restored by additional oleate treatment 7 days after induction, *p < 0.05, **p < 0.01, and ***p < 0.001.

To investigate the effects of lipid availability on BMSC osteogenesis, we began by performing osteogenic assays using murine BMSCs isolated from P14 wild-type C57BL/6J mice. Osteogenic medium was prepared using normal FBS (10%), lipid-reduced serum (LRS; 10%), serum deprivation (SD), and LRS with additional oleate (OL, 75 μM), an unsaturated fatty acid, and treated with isolated murine BMSCs. ALP staining was performed on days 5, 7, and 11 after osteogenic induction, and ALP activity was measured to determine the osteogenesis in different mediums (Figures 1A,B). Moreover, an MTT assay was carried out to evaluate the cell viability 7 days after osteogenic induction under different lipid supplements (Figure 1C). Here, we observed significantly decreased ALP activities of BMSCs in LRS or SD medium, and the suppression could be partially rescued by additional supplementation with oleate (Figure 1B). Notably, the cell survival was significantly reduced in SD medium (Figure 1C; *p* < 0.001), indicating that the viability of BMSCs was impaired due to complete deprivation of serum supplement. Further *in vitro* analysis revealed a significant decrease in protein expression of osteogenic markers such as COL1, RUNX2, and beta-catenin, serving as an intracellular Wnt signaling transducer (Figures 1D,E) 7 days post-induction. We then asked whether the suppressed osteogenesis was also related to the downregulation of Wnt/beta-catenin signaling. Relative mRNA expression of several Wnt target genes (*Tcf1*, *Lef1*, *Axin2*) and osteogenic genes (*Alpl*, *Sp7*, *Col1a1*) was examined and turned out to be reduced significantly in qRT-PCR assays 7 days after induction (Figure 1F). Notably, the suppressed expressions of protein and mRNA of both osteogenesis and Wnt/beta-catenin signaling were rescued by oleate supplementation (Figures 1A–F).

### The Suppressed Osteogenic Differentiation Was Rescued by Oleate Supplementation in a Dosage-Dependent Manner

**FIGURE 2:**
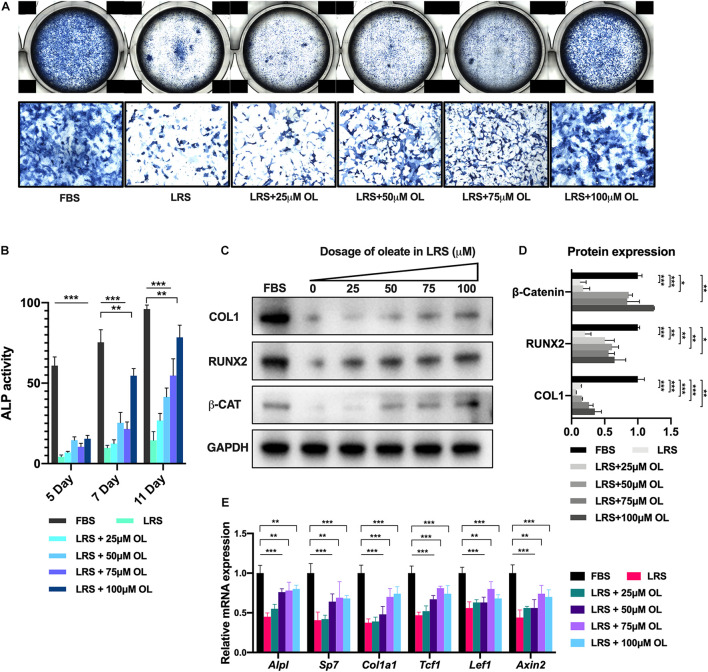
The suppressed osteogenesis was rescued by oleate supplementation in a dosage-dependent manner. **(A)** Alkaline phosphatase (ALP) staining of bone marrow mesenchymal stromal cells (BMSCs) 7 days after osteogenic induction in culture medium with normal fetal bovine serum (FBS; 10%), lipid-reduced serum (LRS; 10%), and LRS (10%) with increasing dosages of oleate (25, 50, 75, 100 μM). **(B)** ALP activity of BMSCs 5, 7, and 11 days after osteogenic induction in different culture mediums. **(C,D)** Western blots showed that the suppressed protein expression levels of osteogenic markers COL1 and RUNX2 and beta-catenin by lipid deprivation were restored by additional oleate treatment in a dosage-dependent manner. **(E)** qRT-PCR revealed that the suppressed mRNA expression levels of osteogenic genes *Alpl*, *Sp7*, and *Col1a1* and Wnt target genes *Tcf1*, *Lef1*, and *Axin2* by lipid deprivation were restored by additional oleate treatment in a dosage-dependent manner, *p < 0.05, **p < 0.01, and ***p < 0.001.

To further validate the rescue effect of oleate supplementation in lipid-deprived BMSCs, we performed an osteogenic assay of BMSCs using increasing dosage of oleate (0, 25, 50, 75, and 100 μM, respectively) in LRS medium for 5, 7, and 11 days (Figure 2A). Quantification of ALP activity revealed that the enhancement of osteogenesis was dosage-dependent on oleate supplementation (Figure 2B). Moreover, the protein and mRNA expressions of osteogenic markers and Wnt target genes were also increased dependent on oleate treatment 7 days after induction (Figures 2C–E). These results suggested that lipid availability could modulate osteogenic differentiation *via* Wnt/beta-catenin signaling in BMSCs.

### *Lrp5* Knockout in Limb Mesenchymal Lineage Impaired Bone Formation

**FIGURE 3:**
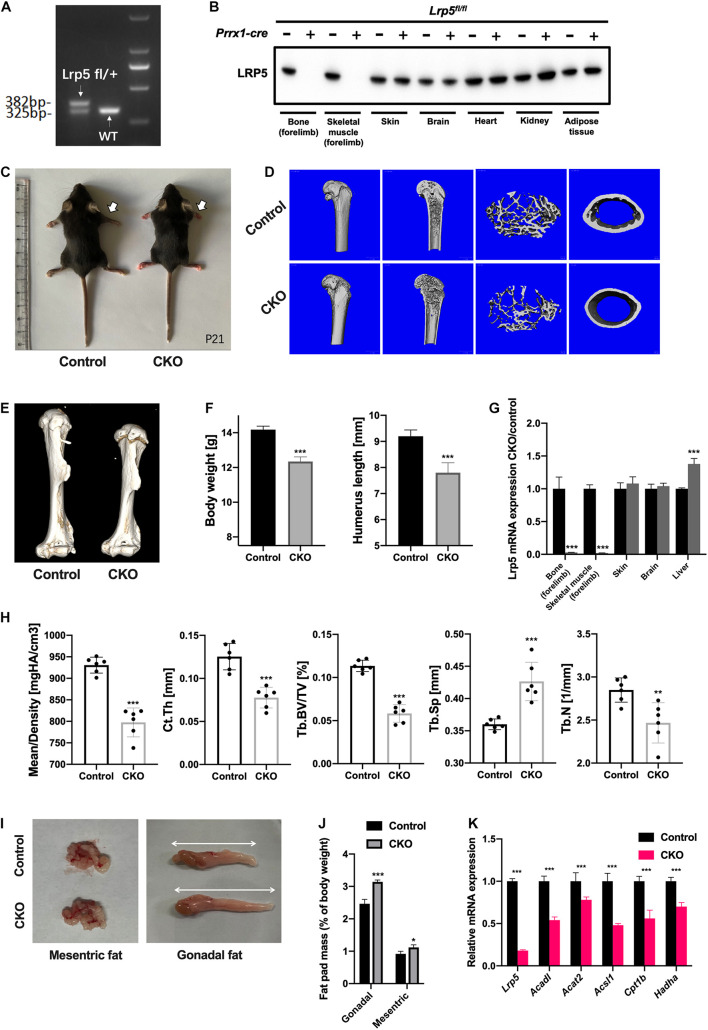
Impaired bone formation and altered adipose tissue distribution in *low-density lipoprotein receptor-related protein 5* (*Lrp5*)*^*fl/fl*^;Prrx1-cre* conditional knockout (CKO) mice. **(A)** Electrophoresis of PCR products to identify *Lrp5*-flox allele in heterozygous (left band) and wild-type (WT) (right band) samples. The band size for WT allele was ∼325 bp and floxed allele was ∼382 bp. **(B)** Protein expression of LRP5 in different organs collected from postnatal day 0 (P0) control (*Prrx1-cre* negative) and CKO (*Prrx1-cre* positive) mice. The LRP5 expression was significantly abrupted in bone and skeletal muscles from forelimb. **(C)** Gross appearance of P21 mice. White arrows indicate shortened forelimbs in CKO (right) mice compared to control (left) mice. **(D)** Representative three-dimensional micro-computed tomography (micro-CT) images of trabecular bony architecture and cortical bone thickness of distal femur harvested from P21 control and CKO mice. **(E)** Representative three-dimensional micro-CT images of humerus harvested from P21 control and CKO mice. **(F)** Body weight and humerus length of CKO mice were quantified (*n* = 6, mean ± standard deviation). **(G)** qRT-PCR analysis revealed abruptions of *Lrp5* gene expression in bone and skeletal tissues harvested from P0 CKO mice. **(H)** Quantified micro-CT data. Data are shown as mean ± standard deviation (*n* = 6). **(I)** Gross appearance of dissected mesenteric and gonadal fat from 2-month-old control and CKO mice. White arrows indicate an increase in the volume of gonadal fat in CKO mice. **(J)** Quantification on mesenteric and gonadal fat pad mass as a percentage of body weight in 2-month-old control and CKO mice. The mesenteric and gonadal fat were significantly increased in CKO mice (*n* = 6, mean ± standard deviation). **(K)** qRT-PCR analysis on bone marrow mesenchymal stromal cells (BMSCs) harvested from mouse forelimbs indicated a significant decrease in mRNA expression levels of lipid metabolism genes, including *Acadl*, *Acat2*, *Acsl1*, *Cpt1b*, and *Hadha*, as well as *Lrp5*, *p < 0.05, **p < 0.01, and ***p < 0.001.

As an important co-receptor for Wnt/beta-catenin signaling and potential endocytic mediator of multiple substances (Li et al., 2001; Schneider and Nimpf, 2003; Chung and Wasan, 2004), LRP5 was previously reported essential in bone development (Clement-Lacroix et al., 2005; Yadav and Ducy, 2010; Chin et al., 2015; Frey et al., 2015; He et al., 2020) and lipid metabolism (Magoori et al., 2003; Loh et al., 2015). We generated CKO mice by crossing *Lrp5*-floxed ready mice with *Prrx1-cre* mouse line to inactivate *Lrp5* gene in limb mesenchymal cell lineage, including BMSCs. The loxP sequence was inserted into the murine genome to conditionally remove the exon 2 of *Lrp5* gene in Cre-expressing lineages. We collected the genome DNA from P10 mouse tail, and a PCR program and the following electrophoresis were performed to determine the floxed *Lrp5* allele (Figure 3A). To further validate the efficiency of Cre-mediated knockout *in vivo*, we harvested multiple organs from control (*Lrp5*^*fl/fl*^) and CKO (*Lrp5^*fl/fl*^; Prrx1-cre*) mice at P0, including bone and skeletal muscles in forelimbs, skin, brain, heart, kidney, and subcutaneous adipose tissue. The following Western blot and qRT-PCR analysis confirmed that the LRP5 protein expression and *Lrp5* mRNA expression were lost in CKO mice in bone and skeletal muscles of forelimbs compared to control mice (Figures 3B,G; all *p* < 0.001). It turned out that the mRNA expression of *Lrp5* was significantly increased in liver tissue (*p* < 0.001), indicating a compensatory enhancement of gene expression in liver. In addition, in P21, the CKO mice presented a reduced body size and shortened limbs in gross appearance (Figure 3C). Besides, the body weight was decreased in CKO mice significantly (Figure 3F; *p* < 0.001).

To determine the effect of *Lrp5* knockout *in vivo*, we performed micro-CT scan on the humerus and femur samples harvested from P21 mice. The humerus length was remarkably decreased in CKO mice (Figures 3E,F; *p* < 0.001). The reconstruction imaging of distal femur recorded decreased density in trabecular architecture and thinner cortical bone in CKO compared to control mice (Figure 3D). Further analysis revealed a significant decrease in mean bone density (*p* < 0.001), average Ct.Th. (*p* < 0.001), Tb.BV/TV (*p* < 0.001), and Tb.N (*p* < 0.01) and increased Tb.Sp (*p* < 0.001) in the distal femur of CKO mice. These data indicated that the bone formation in limb was impaired as a result of suppressed osteogenesis differentiation of skeletal progenitors, including BMSCs by *Lrp5* knockout.

### *Lrp5* Knockout in Bone Marrow Mesenchymal Stromal Cells Altered Fat Distribution in Mice

Previous report showed the lipid metabolism and adipose tissue were changed after *Lrp5* gene was knockout in osteoblast lineage (Frey et al., 2015), indicating the role of LRP5 in modulating lipid availability in skeletal progenitors. In order to test the similar regulatory effect in BMSC-CKO mice, we dissected the adipose tissue from control and CKO mice at 2 months old. Interestingly, there was increased fat pad mass in gonadal (*p* < 0.001) and mesenteric areas (*p* < 0.05; Figures 3I,J), but the subcutaneous fat in limb was not changed (data not shown). In addition, the expression of lipid metabolic genes (*Acadl*, *Acat2*, *Acsl1*, *Cpt1b*, and *Hadha*) in limb BMSCs was significantly decreased (Figure 3K; all *p* < 0.001). There data suggested that the ability of using lipid substances was impaired in BMSCs after *Lrp5* knockout and the accumulation of adipose tissue was increased in mesenteric and gonadal regions, strongly indicating the role of LRP5 in lipid uptake and metabolism in BMSCs.

### *Lrp5* Ablation Suppressed Lipid Uptake and Osteogenic Differentiation of Bone Marrow Mesenchymal Stromal Cells

**FIGURE 4:**
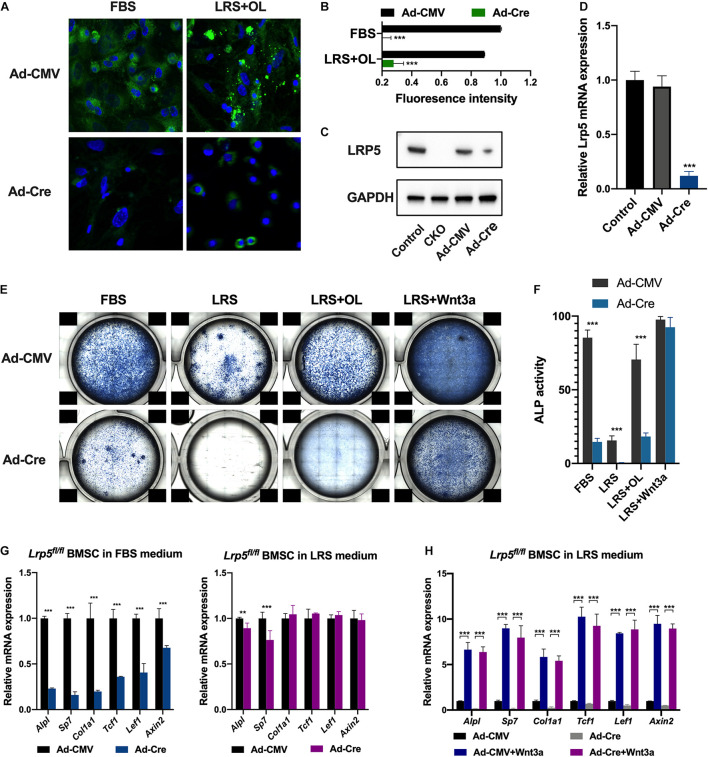
*Low-density lipoprotein receptor-related protein 5 (Lrp5*) ablation in bone marrow mesenchymal stromal cells (BMSCs) impairs lipid uptake and osteogenesis. **(A)** BODIPY^TM^ staining revealed significantly decreased lipid droplets in BMSC cytoplasm after *Lrp5* ablation using adenoviral Cre-recombinase. **(B)** Quantified fluorescence intensity of lipid droplets in BMSC cytoplasm indicating impaired lipid uptake of *Lrp5*-KO BMSCs. **(C)** LRP5 protein expression in BMSCs harvested from postnatal day 0 (P0) control mice, conditional knockout (CKO) mice, *Lrp5*-control BMSC, and *Lrp5*-KO BMSCs. **(D)** qRT-PCR validated the knockout effect of adenoviral-Cre infection on *Lrp5*^*fl/fl*^ BMSCs. **(E)** Alkaline phosphatase (ALP) staining of control and *Lrp5*-KO BMSCs 7 days after osteogenic induction in culture medium with normal fetal bovine serum (FBS), lipid-reduced serum (LRS), LRS with 100 μM oleate treatment, and LRS with recombinant 100 ng/ml Wnt3a treatment, respectively. **(F)** ALP activity of BMSCs 7 days after osteogenic induction in different culture mediums. **(G)** mRNA expression levels in control and *Lrp5*-KO BMSCs of osteogenic genes *Alpl*, *Sp7*, and *Col1a1* and Wnt target genes *Tcf1*, *Lef1*, and *Axin2* after 7 days of osteogenic induction in FBS or LRS culture medium. **(H)** mRNA expression levels in control and *Lrp5*-KO BMSCs of osteogenic genes *Alpl*, *Sp7*, and *Col1a1* as well as Wnt target genes *Tcf1*, *Lef1*, and *Axin2* after 7 days of induction in LRS culture medium with or without Wnt3a treatment, *p < 0.05, **p < 0.01, and ***p < 0.001.

To further understand the role of LRP5 in lipid uptake of BMSCs, we used adenovirus carrying CMV-Cre-recombinase (Ad-Cre) or CMV-null (Ad-CMV) expressing vector to infect BMSCs isolated from P14 *Lrp5*^*fl/fl*^ mice. The expression of LRP5 at protein and mRNA levels was examined to validate the efficiency of Cre-recombination (Figures 4C,D; *p* < 0.001). To trace the endocytosis of lipids *in vitro*, we applied BODIPY^TM^ staining (DiDonato and Brasaemle, 2003) in FBS and LRS + OL culture condition 48 h after viral infection (Figure 4A). The fluorescence intensity of lipid droplet signals in cytoplasm was found significantly decreased after *Lrp5* ablation both in FBS and LRS + OL conditions (Figure 4B; both *p* < 0.001), suggesting impaired endocytosis of lipid due to *Lrp5* ablation. We next performed osteogenic assays on adenoviral-treated BMSCs in FBS and LRS mediums (Figure 4E). Consistent with *in vivo* results, the osteogenesis of BMSCs was significantly inhibited by *Lrp5* ablation (Figures 4E,F; all *p* < 0.001) after 7-day induction in both assays, indicating the modulatory role of LRP5 in BMSC osteogenesis.

### The Wnt-Activating Osteogenesis Was Independent of Lipid Scarcity

In light of the fact that Wnt-LRP5 signaling was found to participate in the lipid metabolism and bone formation in skeletal progenitors, we then examined whether this modulation is regulated by lipid availability or not. After 7-day osteogenic induction, *Lrp5* knockout significantly reduced the expression of Wnt target genes (*Tcf1*, *Lef1*, *Axin2*; Figure 4G; all *p* < 0.001), as well as osteogenic genes (*Alpl*, *Sp7*, *Col1a1*; Figure 4G; all *p* < 0.001) in FBS-treated BMSCs, which was consistent with previous reports (Ayturk et al., 2013; Sebastian et al., 2017). However, the changes in Wnt target gene expression were not significant after *Lrp5* ablation in lipid deprivation medium (Figure 4G). These data suggested that the modulation of LRP5 in BMSC osteogenesis was mediated by Wnt/beta-catenin signaling and was dependent on lipid availability. We further used recombinant Wnt3a protein, a classic Wnt ligand, in the lipid-deprived BMSCs with/without *Lrp5* ablation (Figure 4E). The osteogenic activity was significantly enhanced even compared with *Lrp5*-intact BMSCs (*Lrp5*^*fl/fl*^ treated with Ad-CMV) in FBS medium (Figures 4E,F; *p* < 0.001). These results indicated that the Wnt-activating osteogenesis was independent of lipid scarcity.

## Discussion

Lipid metabolism dysfunction, including atherosclerosis and hypercholesterolemia, was considered to be related to osteoporosis in many clinical studies (Yamaguchi et al., 2002; Buizert et al., 2009; Lampropoulos et al., 2012), shedding light on the dynamics of mesenchymal stromal cell lineage differentiation and specification (Loh et al., 2015; Yue et al., 2016; van Gastel et al., 2020). Wnt/beta-catenin signaling and its components play essential roles in bone homeostasis and lipid metabolism, while the molecular underpinnings of their regulations remain to be clarified. Here, we showed that lipid scarcity suppressed osteogenic differentiation of BMSCs and Wnt/beta-catenin signaling *in vitro*, and the suppression could be rescued by additional fatty acid supplementation in a dosage-dependent manner. We also demonstrated that the Wnt co-receptor, LRP5, participated in lipid uptake of BMSCs and modulated bone formation and lipid metabolism *in vitro* and *in vivo*.

Lipid depots in bone marrow area can be mobilized into fatty acids and serve as one of the chief fuel sources for bone-residing cells and BMSCs. Recent studies uncovered further essential roles of lipids in bone marrow and other skeletal niche in bone development and functioning (Rendina-Ruedy and Rosen, 2020). Most recently, van Gastel et al. (2020) reported that the lipids in microenvironment were responsible for the specification of multiple skeletal progenitor lineages during bone-healing process. In the study, they recorded that when lipids were scarce, skeletal progenitors preferentially underwent chondrogenesis rather than osteoblastogenesis due to the activated SOX9 signaling and thus adapted to an avascular life. Notably, there are also studies about how lipids modulate mesenchymal stromal cell behaviors (Clemot et al., 2020; Senos Demarco et al., 2020). Another study reported that adipose-derived MSCs could reciprocally modulate lipid metabolism *via* reprogramming macrophages (Souza-Moreira et al., 2019). In the present study, we investigated the impact of lipid scarcity on BMSC osteogenesis *in vitro*. Deprivation of lipid from serum supplement significantly impaired the osteogenic capacity of BMSCs (Figures 1A,B) and suppressed Wnt/beta-catenin signaling (Figures 1D,E). Moreover, additional supplementation with fatty acid, oleate, was able to restore the osteogenesis deprived by lipid scarcity in a dosage-dependent manner (Figures 2A,B). These data suggested that lipids mediated BMSC differentiation by providing fatty acids as sources for oxidation (FAO). The block of FAO would potentially inhibit osteogenesis and activation of Wnt/beta-catenin signaling in BMSC lineages.

As a member of LDL receptor family, LRP5 serves as a transmembrane co-receptor for canonical Wnt/beta-catenin signaling pathway and plays an essential role in bone development and homeostasis (Cui et al., 2011; Baron and Kneissel, 2013; Kobayashi et al., 2016). Mutations in *LRP5* genes are responsible for a spectrum of skeletal phenotypes in human, ranging from low to high bone density (Gong et al., 2001; Van Wesenbeeck et al., 2003; Levasseur et al., 2005; Baron and Kneissel, 2013). The past decade had witnessed frequent breaks in therapeutic development targeting LRP5 signaling, namely, the anti-DKK1 and anti-sclerostin molecules in the treatment of postmenopausal osteoporosis (Li et al., 2005; Niehrs, 2006; Williams, 2017). LRP5 was also observed to be involved in lipid metabolism. In human, gain-of-function *LRP5* mutations led to higher bone mass and lower body fat accumulation, while a low bone mineral density-associated common LRP5 allele correlated with increased abdominal adiposity (Loh et al., 2015). Similarly, LRP5 deficiency led to increased plasma cholesterol levels in mice fed a high-fat diet caused by the decreased hepatic clearance of chylomicron remnant. Meanwhile, LRP5-deficient mice showed a markedly impaired glucose tolerance (Loh et al., 2015). Magoori et al. (2003) used apoE;*Lrp5* double-knockout mice to investigate the role of LRP5 in lipoprotein metabolism and found severe hypercholesterolemia, impaired fat tolerance, and increased atherosclerosis in DKO mice. Previously, Wnt-LRP5 signaling was found to regulate fatty acid oxidation in osteoblasts (Frey et al., 2015). By using *Osx-cre*, researchers also recorded a decrease in bone mass and increase in body fat for CKO mice. In the present study, we generated BMSC CKO mice using *Prrx1-cre* and provided strong evidence for LRP5 participating in the regulation of osteogenesis and lipid uptake of BMSCs. The CKO mice developed less bone formation (Figures 3D–H) and increased adipose tissue in mesenteric and gonadal regions (Figures 3I,K). Lipid droplets were retained in BMSC cytoplasm after *Lrp5* ablation and the osteogenesis was inhibited (Figures 4A–F). Taken together, our data suggested that the loss of LRP5 in BMSC impaired the capacity to uptake and use lipid substances during osteogenesis, which explained the phenotypes of reduced bone quality and accumulated fat mass *in vivo*.

The canonical Wnt/beta-catenin signaling pathway is known as a key regulator of bone development, metabolism, and homeostasis (Niehrs, 2012; Kobayashi et al., 2016). The co-receptors LRP5/6 respond to Wnt ligands including Wnt1, Wnt3a, and Wnt10b to regulate bone formation (Boland et al., 2004; Bennett et al., 2007; Keupp et al., 2013). However, previous study revealed Wnt3a preferentially acted *via* LRP6 in osteoblasts, and LRP5 played a less significant role in mediating Wnt signaling (Sebastian et al., 2017). Another study recorded that lithium chloride activates canonical Wnt/beta-catenin signaling in cultured *Lrp5*^–/–^ osteoblasts and *in vivo*, also indicating an LRP5-independent way to activate Wnt signaling (Clement-Lacroix et al., 2005). Additionally, duodenum-derived serotonin was found to regulate bone formation in an LRP5-dependent manner (Yadav et al., 2008), suggesting a more complicated role of LRP5 in bone development. In the present study, we found that the regulation of lipids on BMSC osteogenesis was also LRP5-dependent, as *Lrp5* ablation in normal FBS treatment significantly impairs ALP activity of BMSCs (Figure 4E, panel 1). Moreover, Wnt3a activated canonical Wnt/beta-catenin signaling and BMSC osteogenesis *via* LRP5-independent manner, as the ALP activity and Wnt downstream targets were significantly enhanced by Wnt3a treatment in *Lrp5*-KO BMSCs (Figures 4E, panels 3, 4; H). These data provided further evidence in Wnt-LRP5 regulatory network in bone formation and other biological processes.

In summary, we investigated the influence of lipid scarcity on BMSC differentiation and provided more insight to the role of LRP5 in mediating lipid uptake in BMSC osteogenic differentiation and metabolism. These results further unveil the critical role of lipid availability in the biological functions of mesenchymal progenitors. Clinically, LRP5 functions essentially in bone mass and lipid metabolism in osteoporosis or cardiovascular patients. Our data suggested novel therapeutic discoveries targeting LRP5 in the treatment of bone disorders and metabolic diseases.

## Conclusion

Our study uncovered the regulatory role of LRP5 and lipids in osteogenic differentiation of BMSCs. Lipid scarcity suppressed osteogenic differentiation of BMSCs and Wnt/beta-catenin signaling. LRP5 acted as mediators in lipid uptake of BMSCs and thus modulated osteogenesis and lipid metabolism both *in vitro* and *in vivo*. As a valuable therapeutic target in the treatment of osteoporosis and cardiometabolic disorders, the biological functions and molecular implications of Wnt-LRP5 regulations require further investigation.

## Data Availability Statement

The original contributions presented in the study are included in the article/Supplementary Material, further inquiries can be directed to the corresponding author.

## Ethics Statement

The animal study was reviewed and approved by the Ethics Committee of Peking Union Medical College Hospital.

## Author Contributions

JcL and ZW conceptualized and designed the study. ZW and GQ were responsible for the administrative and funding supports. JcL conducted investigations on animals and cell lines and wrote the original draft. ZZ and GY were responsible for the collection and assembly of data. JyL and JcL were responsible for the data analysis and interpretation. ZW, LL, and HW were responsible for the review and editing the final manuscript. All authors made the final approval of the article.

## Conflict of Interest

GY is employed by Harmony Technology Co., Ltd. (Beijing, China). The remaining authors declare that the research was conducted in the absence of any commercial or financial relationships that could be construed as a potential conflict of interest.

## Publisher’s Note

All claims expressed in this article are solely those of the authors and do not necessarily represent those of their affiliated organizations, or those of the publisher, the editors and the reviewers. Any product that may be evaluated in this article, or claim that may be made by its manufacturer, is not guaranteed or endorsed by the publisher.

## Footnotes

1
https://imagej.nih.gov/ij/
